# Artificial intelligence in postural management: a critical review of detection, correction, and clinical applicability

**DOI:** 10.1186/s13018-025-06640-z

**Published:** 2026-01-21

**Authors:** Yaşar Köroglu, Elham Hosseini, Ziya Bahadır, Bayram Karakus, Mohammad Alimoradi, Mohammad Alghosi, Andreas Konrad

**Affiliations:** 1 Faculty of Sports Sciences, Sivas Cumhuriyet University, Sivas, Turkey; 2 Department of Sports Injuries and Corrective Exercises, Faculty of Sports Sciences, Shahid Bahonar University of Kerman, Kerman, Iran; 3 Department of Physical Education and Sports, Institute of Health Sciences, Erciyes University, Kayseri, Turkey; 4 HERC- Health, Exercise and Research Center, Mina Rashid, Dubai Maritime City, Dubai, UAE; 5 Department of Physical Education, Technical and Vocational University (TVU), Tehran, Iran; 6 Institute of Human Movement Science, Sport and Health, Graz University, Graz, Austria

**Keywords:** Artificial intelligence, Postural correction, Human pose estimation, Tele-rehabilitation, Musculoskeletal disorders

## Abstract

**Background:**

Poor posture and related musculoskeletal conditions represent a growing global health concern. Conventional postural assessment methods are often subjective, intermittent, and insufficient for accurate, continuous monitoring. Advances in artificial intelligence (AI), particularly in computer vision and human pose estimation (HPE), have introduced new possibilities for objective and real-time postural analysis.

**Main body:**

This critical review synthesizes and evaluates current developments in AI technologies for postural management. The review draws on recent literature from computer science, bioengineering, and clinical research, focusing on studies from the past decade that explore the use of AI and HPE in the detection, monitoring, and correction of human posture. AI-based HPE models demonstrate high precision in identifying anatomical landmarks and quantifying postural parameters, offering a robust alternative to traditional assessment methods. Applications are expanding beyond laboratory environments to practical contexts such as ergonomic risk evaluation and sports performance analysis. In addition, AI-driven systems that deliver real-time feedback and support tele-rehabilitation are enhancing user engagement and enabling personalized interventions. Despite these advancements, the field faces several challenges. Evidence from large-scale clinical trials remains limited, and the generalizability of existing models across diverse populations and real-world conditions is uncertain. Concerns related to usability, data privacy, and integration within healthcare systems also pose significant barriers to clinical translation.

**Conclusion:**

AI holds considerable potential to transform postural management through continuous, objective, and accessible assessment and intervention. To fully realize this potential, future work must extend beyond technical innovation to include rigorous clinical validation, user-centered design, and the establishment of ethical and regulatory frameworks that ensure safe, effective, and equitable implementation.

## Background

Musculoskeletal disorders (MSDs) associated with poor posture represent a pervasive public health challenge and impose a significant burden on individuals and healthcare systems worldwide [1]. Common conditions such as chronic low back pain, neck and shoulder strain, and spinal deformities are frequently linked to prolonged non-neutral postures exacerbated by sedentary lifestyles, occupational demands, and reduced physical activity [2–4]. Effective postural management is crucial for preventing these conditions, mitigating their progression, and supporting rehabilitation. This approach encompasses accurate detection, continuous monitoring, and timely corrective intervention [1, 4, 5].

In physiotherapy and ergonomics, the primary goals of postural correction are to restore biomechanical alignment, reduce tissue loading, and improve functional capacity and quality of life [5, 6]. For decades, clinicians have relied on conventional postural assessment methods including visual inspection, plumb lines, goniometers, and intermittent imaging such as radiographs [6–8]. Although widely used, these methods have fundamental limitations. Visual observation is inherently subjective and suffers from low inter- and intra-rater reliability [9, 10]. Manual measurements are labor-intensive capture only isolated snapshots of posture, and typically fail to represent dynamic or habitual postural behaviors during daily activities [8–10]. Radiographic imaging, while anatomically precise, is unsuitable for routine monitoring due to radiation exposure and its static nature [10, 11]. Most importantly, traditional techniques cannot support continuous, objective, and real-time corrective feedback, which is critical for motor relearning and long-term behavioral change. These shortcomings limit the effectiveness of conventional interventions and underscore the need for more precise, scalable, and interactive technologies.

In response to these gaps, the last decade has witnessed rapid progress in artificial intelligence (AI), particularly within computer vision (CV) and human pose estimation (HPE), which has fundamentally transformed the possibilities for postural assessment and correction [12–14]. HPE algorithms localize anatomical key points (e.g., shoulders, hips, knees) to reconstruct 2D or 3D skeletal representations of the human body, enabling detailed quantitative analysis of posture and movement [13–15]. Deep learning–based HPE models have achieved substantial improvements in accuracy, robustness, and generalizability, allowing them to detect subtle postural deviations that are often imperceptible to the human eye [17–19]. Modern models can now operate in real time on consumer-grade devices, extending postural analysis beyond controlled laboratory settings into homes, workplaces, and clinical environments [16, 18, 20].

These technological advances have enabled a new generation of active postural corrective systems, addressing limitations inherent to conventional approaches. AI-driven platforms can continuously monitor posture and deliver real-time biofeedback through visual, auditory, or haptic cues, prompting immediate correction when deviations occur [21–24]. This closed-loop feedback mechanism enhances proprioception, reinforces correct alignment, and supports motor learning—key factors for reducing maladaptive patterns associated with MSDs. AI-based systems can also analyze dynamic tasks, detect compensatory movements, and provide exercise-specific feedback, making them highly effective for injury prevention and for correcting movement patterns during rehabilitation [19, 20, 22].

**Fig. 1:**
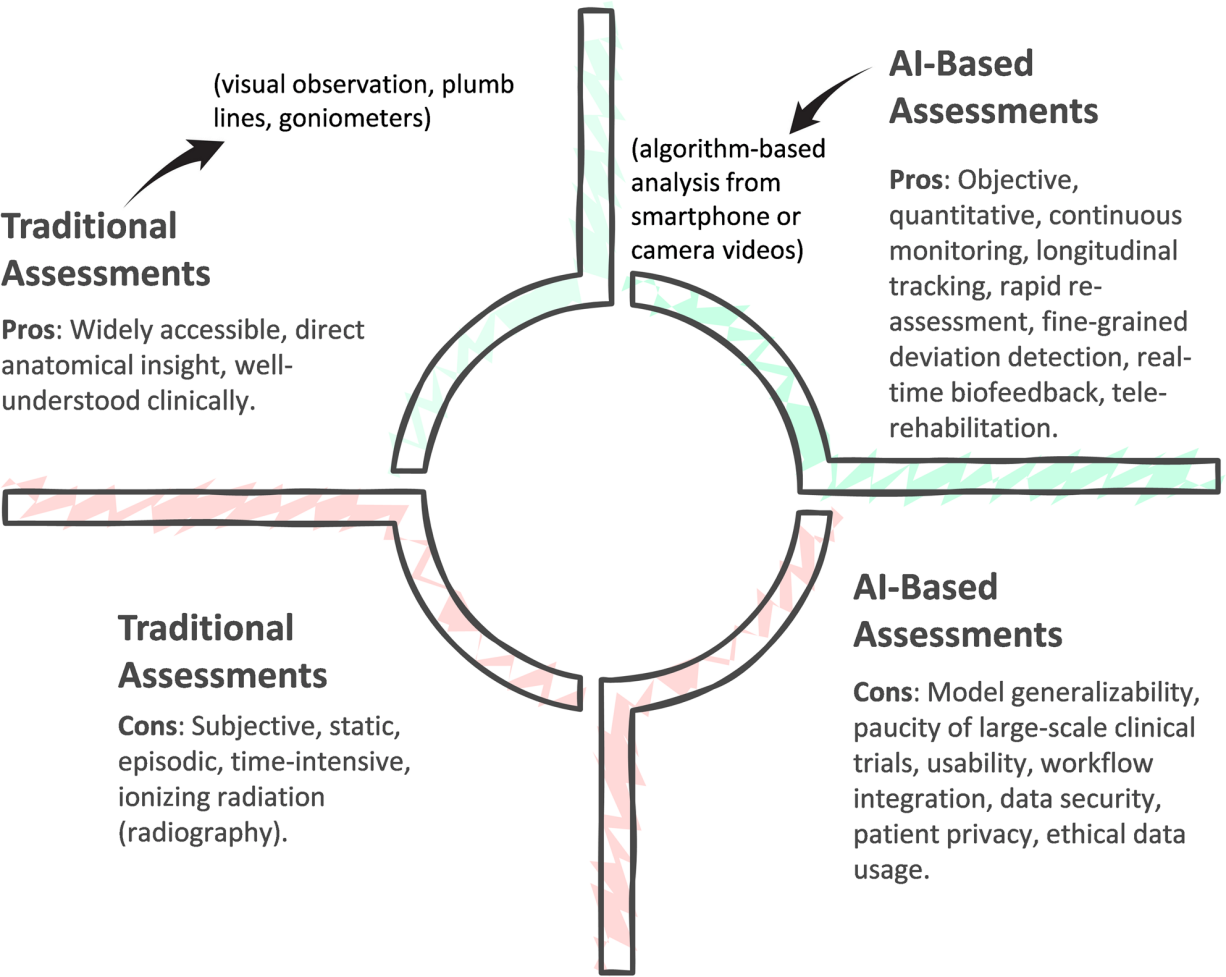
Traditional versus AI-based postural assessment

Moreover, the integration of AI with tele-rehabilitation (TR) has expanded access to physiotherapy and enhanced the quality of remote care. TR platforms empowered by AI and HPE can objectively assess posture and movement via standard cameras or sensors, guide patients through therapeutic exercises, track adherence, and automatically adjust difficulty based on performance [24, 25]. Advances in high-performance computation, particularly the development of lightweight deep networks for edge devices, have enabled these AI tools to run efficiently on smartphones, tablets, and low-cost cameras, greatly increasing their scalability and clinical reach [26]. A comparative overview of traditional and AI-based postural assessment paradigms is illustrated in Fig. 1.

Despite the immense technological promise and burgeoning research activity, a critical disconnect exists between the demonstrated capabilities of AI in controlled environments and its proven value in routine clinical practice. The literature is replete with studies reporting high technical accuracy for posture detection models, yet there remains a significant scarcity of rigorous, large-scale clinical trials that validate the long-term efficacy of these systems in improving patient outcomes compared to standard care [26, 27]. Issues of generalizability are paramount; a model trained on a limited dataset may not perform reliably across diverse populations, body types, clothing, and real-world environments. Moreover, the practical integration of these technologies into complex clinical workflows presents substantial challenges related to usability, clinician training, data security, patient privacy, and regulatory approval.

Therefore, a critical review is necessary to cut through the technological hype and provide a balanced perspective on the current state and future trajectory of AI in postural management [17, 18, 28, 29]. Hence, this review aims to synthesize and critically analyze the existing body of knowledge across three fundamental pillars. First, it will examine the core AI technologies and data acquisition modalities used for postural detection, evaluating their accuracy and performance benchmarks. Second, it will assess the evidence for the efficacy of AI-based postural correction interventions, including real-time biofeedback and TR programs. Ultimately, it will examine the critical challenges and considerations for clinical applicability, including usability, healthcare system integration, and the ethical implications of responsible innovation. By providing a comprehensive overview of both the opportunities and obstacles, this review aims to inform future research, guide clinical implementation, and foster a collaborative dialogue among technologists, clinicians, and policymakers to responsibly harness the transformative potential of AI in improving human postural health.

## Methodological approach for the review

To comprehensively address the multifaceted and rapidly evolving landscape of AI in postural management, this study employs a critical narrative review methodology. Unlike a systematic review, which adheres to a rigid protocol to answer a narrowly focused clinical question, a critical narrative review is better suited for synthesizing information from a broad and heterogeneous body of literature spanning multiple disciplines, including computer science, bioengineering, and clinical medicine [30]. This approach enables a more flexible and interpretive synthesis, allowing for the identification of dominant themes, a critical analysis of underlying assumptions, and the charting of future research directions in a nascent field. The objective is not to perform a meta-analysis of quantitative data, which would be premature given the diversity of AI models and evaluation metrics, but rather to construct a coherent and critical narrative on the current state of technology, evidence for its application, and the challenges impeding its translation into practice [30, 31]. This mirrors approaches used to provide a comprehensive analysis of ML models in related fields, such as human movement analysis [28].

The foundation of this review is a comprehensive exploration of scientific literature. The search was strategically designed to capture the significant advancements in the field over the last decade (2014–2025), a period characterized by the ascent of DL and its profound impact on CV and human pose estimation. A non-exhaustive search was conducted in major academic databases, including PubMed, IEEE Xplore, ACM Digital Library, and Google Scholar, to identify relevant publications. The search strategy was concept-based, combining keywords related to the core technology, the application domain, and the clinical context to ensure broad coverage of the interdisciplinary landscape. Key search terms were organized into three main categories. The first category targeted AI and CV technologies, using terms such as “artificial intelligence”, “machine learning”, “deep learning”, “computer vision”, and “human pose estimation”. The second category focused on the application domain of posture, incorporating keywords like “postural assessment”, “posture analysis”, “postural correction”, “spinal alignment”, “ergonomics”, and “biomechanics”. The third category encompassed clinical and interventional contexts, including terms such as “musculoskeletal disorders”, “physiotherapy”, “rehabilitation”, “biofeedback”, and “tele-rehabilitation”. Boolean operators (AND, OR) were used to combine these terms in various permutations to retrieve a comprehensive set of potentially relevant articles. The reference lists of retrieved articles and relevant review papers were also manually screened to identify additional studies.

The selection of literature for inclusion was guided by thematic relevance. We included peer-reviewed journal articles and systematic reviews that directly addressed the application of AI or ML for the detection, monitoring, or correction of human posture. Studies were drawn from both technical and clinical domains to ensure a holistic perspective, covering the spectrum from algorithmic development and performance benchmarking to clinical application and evaluation. The review encompassed a variety of data acquisition modalities, including vision-based systems and wearable sensors, when their data was processed using AI algorithms. This broad scope is essential for understanding the full range of technological solutions being explored for postural management, from sports biomechanics to rehabilitation platforms. Articles were excluded if they did not primarily focus on AI-driven approaches, were concerned with non-human subjects, or addressed applications unrelated to human health, ergonomics, or rehabilitation. Editorials, abstracts without full-text availability, and non-peer-reviewed sources were also excluded to maintain the academic rigor of the review. This process ensured that the included literature formed a robust and relevant basis for a critical analysis of the field.

The synthesis of the selected literature was conducted through a thematic analysis framework. After an initial screening of titles and abstracts, full-text articles were retrieved and reviewed in detail. Data and key concepts were extracted and organized according to a predefined structure that forms the backbone of this review. This structure is aligned with the primary objectives and comprises three overarching themes: (1) a critical analysis of AI technologies for postural detection, focusing on core models, data acquisition, and performance benchmarks; (2) an evaluation of the efficacy of AI-based postural correction interventions, such as real-time biofeedback and TR programs; and (3) an exploration of clinical applicability, integration challenges, and ethical considerations. This thematic approach facilitates a coherent narrative, enabling the juxtaposition of technological advancements with clinical realities and the critical evaluation of evidence supporting various applications. By systematically organizing the findings, this review aims to provide a clear and structured overview of the field's achievements, limitations, and the critical roadmap for future research and development.

## A critical analysis of AI technologies for postural detection

Recent advancements in AI, especially in CV, have transformed postural detection from a qualitative art into a quantitative science, enabling objective, continuous, and accessible assessment [18, 28, 29]. This chapter provides a critical analysis of the AI technologies that underpin modern postural detection systems. It examines the core AI models for HPE, evaluates the various data acquisition modalities and processing pipelines, and benchmarks the accuracy and clinical performance of these emerging systems, laying the groundwork for understanding their utility in corrective interventions and clinical practice.

## Core AI models and algorithms in posture recognition

At the heart of AI-driven postural detection lies HPE, a fundamental CV task focused on identifying the configuration of a human body in an image or video [14, 18, 32, 33]. The primary objective of HPE is to localize anatomical key points to create a skeletal representation of the body [32, 34], which serves as the foundational data structure for any subsequent biomechanical analysis [35, 36]. The evolution of HPE models, particularly the transition from classical ML approaches to modern DL-based architectures, has been a principal catalyst for the recent surge in automated postural assessment tools [14, 33, 37]. Early HPE methods relied on handcrafted features (e.g., Histogram of Oriented Gradients) and probabilistic graphical models (e.g., Pictorial Structures) to infer body part locations [12, 18]. While innovative for their time, these models were brittle, struggling with variations in clothing, lighting, background clutter, and the wide spectrum of human body shapes and poses [18]. The advent of DL, specifically deep convolutional neural networks (CNNs), marked a paradigm shift by enabling models to automatically learn hierarchical feature representations directly from raw pixel data [12, 18, 38]. This end-to-end learning approach led to a dramatic improvement in robustness and precision. The trajectory of DL-based HPE has been characterized by successive architectural innovations, each addressing key limitations of its predecessors [14, 18]. Early CNN architectures like DeepPose treated pose estimation as a regression problem, while the Stacked Hourglass Network introduced a repeated bottom-up (encoding) and top-down (decoding) structure to capture and consolidate information across multiple scales, significantly refining pose estimates [38]. A critical limitation of many early models was the loss of spatial precision due to down sampling. This was directly addressed by architectures like HRNet (High-Resolution Net), which maintains high-resolution representations throughout the entire network process, rather than recovering them later, leading to more precise key point localization essential for measuring subtle postural deviations [37–39]. More recently, the field has seen the integration of transformer architectures, originally dominant in natural language processing. Vision transformers and their hybrids (e.g., Pose Transformer) can capture long-range dependencies between body joints across an image, making them inherently more robust to partial occlusions—a common challenge in real-world clinical and home environments [39, 40]. The evolution of these core models, with their key improvements and impact, is summarized in Table 1. Modern HPE models can be broadly categorized based on the dimensionality of their output: 2D and 3D, a distinction critical for the depth and accuracy of the resulting biomechanical analysis [15, 33, 39].

**Table 1:** Evolution of core AI models for human pose estimation in postural analysis

Model paradigm	Key algorithms and architectures	Key technical improvements
Classical ML and handcrafted features	Pictorial structures, deformable part models	Relied on manually designed features (e.g., HOG, SIFT). Used probabilistic models for spatial relationships
Early DL (2D HPE)	Deep pose, Stacked hourglass networks	Introduced end-to-end learning with CNNs. Hourglass used repeated bottom-up, top-down processing for refinement
Advanced 2D and 3D HPE	2D: HRNet, HigherHRNet, Pose Transformer 3D: VideoPose3D, METRO, SPIN (SMPL)	HRNet: Maintains high-resolution features Transformers: Capture global context 3D Lifting/model-fitting: Recovers accurate 3D pose from 2D or RGB-D data

AI, Artificial intelligence; CNN, Convolutional neural network; DL, Deep learning; HOG, Histogram of oriented gradients; HPE, Human pose estimation; HRNet, High-resolution Net; METRO, Mesh transformer with relative occlusion; ML, Machine learning; RGB-D, Red, green, blue-depth; SIFT, Scale-invariant feature transform; SMPL, Skinned multi-person linear model; SPIN, SMPL oPtimization IN the loop

### 2D human pose estimation

This is the most common form of HPE, where the model predicts the (x, y) coordinates of key points within a two-dimensional image plane. While 2D HPE is computationally less intensive and can be performed using a single, standard RGB camera, its utility for comprehensive biomechanical analysis is limited due to perspective distortion [39]. Despite this, advanced 2D models like HRNet and HigherHRNet are highly effective for identifying gross postural asymmetries and serve as a crucial, accessible component in many TR and wellness applications [32, 39].

### 3D human pose estimation

This advanced form aims to recover the (x, y, z) coordinates of body key points in three-dimensional space, providing a metrically accurate representation essential for precise calculation of joint angles and segment orientations [15, 33, 40]. Achieving 3D HPE involves several technical approaches. Lifting-based methods (e.g., VideoPose3D) first estimate accurate 2D key points and then use a separate network to “lift” them into 3D space, but can struggle with depth ambiguity. Volumetric approaches reason in 3D space directly but are computationally expensive. A particularly promising direction is model-based regression, where algorithms like SPIN (SMPL oPtimization IN the loop) iteratively fit a parametric human body model (e.g., SMPL) to image data, yielding not just a 3D skeleton but a full 3D mesh, enabling a richer biomechanical analysis [40, 41]. These methods can utilize multiple synchronized cameras, specialized depth-sensing cameras, or, increasingly, deduce 3D pose from a single 2D image. The power of these advanced DL models lies in their ability to facilitate the identification of subtle postural deviations that might be missed by the human eye [32, 35]. Furthermore, the development of lightweight and efficient model architectures has made it possible to deploy these systems for real-time analysis on consumer-grade devices, moving postural assessment out of the laboratory and into everyday environments [42]. These core AI models represent a continuous technological evolution, providing the engine for a new generation of tools designed for objective and granular biomechanical posture analysis [33, 35, 36].

## Data acquisition modalities and processing techniques

The performance and clinical utility of an AI-based postural detection system are inextricably linked to the quality and nature of its input data [32, 35]. The choice of data acquisition modality, meaning the hardware used to capture postural information, dictates the type of data available for analysis. It also determines its dimensionality, whether it is 2D or 3D, and its suitability for different application contexts. These contexts range from home-based wellness monitoring to precise clinical diagnostics [14, 32, 37]. Once captured, this raw data must undergo significant processing to be transformed into a format that AI models can interpret and that clinicians can use for biomechanical assessment [32, 36]. The dominant modality in modern postural assessment is vision-based, leveraging cameras and image sensors to capture the human form. This approach is often referred to as *markerless motion analysis* because, unlike traditional laboratory-grade motion capture systems, it does not require subjects to wear reflective markers on their body [32, 35, 36]. This markerless nature dramatically lowers the barrier to entry, making postural analysis more accessible, less intrusive, and easier to deploy in non-clinical settings [32, 36]. Key vision-based modalities include:

### 2D RGB cameras

Standard color cameras, such as those found in smartphones, webcams, and security systems, are the most ubiquitous and cost-effective sensors for postural analysis [13, 35, 36]. Their widespread availability makes them an ideal choice for scalable TR and consumer wellness applications. Systems using 2D cameras typically rely on 2D HPE models to extract postural key points [14, 35]. The primary challenge is translating these 2D data points into clinically meaningful biomechanical insights, which often requires careful camera positioning and standardized protocols to minimize perspective distortion [13, 39, 40].

### Depth-sensing cameras

Devices like the Microsoft Kinect or Intel RealSense represent a step up in capability [34]. These sensors supplement a standard RGB camera with an infrared projector and sensor that can measure depth, creating a “depth map” of the scene [14, 33, 34]. This provides direct 3D information, allowing AI models to perform more robust 3D HPE. By capturing the three-dimensional structure of the body, these systems can more accurately calculate joint angles and body segment parameters, making them well-suited for applications that require higher biomechanical fidelity, such as detailed ergonomic evaluations or tracking rehabilitation progress. Beyond vision-based systems, wearable sensors offer an alternative or complementary approach to data acquisition [32, 33, 35, 36]. Though CV is a major focus, comprehensive systems may integrate data from other sources [36]. Technologies like Inertial measurement units (IMUs), which typically contain an accelerometer, gyroscope, and magnetometer, can be placed on different body segments to directly measure their orientation and movement in 3D space [42, 43]. Although requiring users to wear physical devices, IMU-based systems are unaffected by environmental factors such as poor lighting or occlusion (i.e., when body parts are hidden from a camera’s view), which can pose significant challenges for vision-based methods [35, 43]. In addition, Lighthouse-based systems, such as the HTC Vive Tracker, provide high-accuracy 3D pose estimation using infrared laser sweeps from fixed base stations. Valued in biomechanics for their sub-centimeter precision and relatively low cost, they are effective for full-body motion tracking. Their primary constraints are the need for a calibrated space and a continuous line-of-sight between the trackers and lighthouses [44, 45]. Regardless of the modality, the raw sensor data must be processed before and after AI analysis [32]. The initial stage, pre-processing, involves preparing the data for the HPE model. For images, this may include resizing, cropping, and normalization to match the model’s expected input format [14, 18, 35, 38]. The core processing stage is handled by the AI model itself, which takes the prepared data and outputs the coordinates of the anatomical key points [14, 39]. The final and most clinically relevant stage is post-processing, where these key point coordinates are used to derive meaningful biomechanical metrics [32, 35]. This involves applying biomechanical models to the skeletal data to calculate parameters such as:

- Joint angles (e.g., knee flexion, neck inclination)
- Distances and ratios (e.g., shoulder-hip alignment)
- Kinematic variables (e.g., velocity and acceleration of body segments during movement) [32, 35, 42, 43]

This transformation from raw data to actionable information is critical. It allows the system to assess biomechanical strain, quantify postural asymmetries, and track changes over time [32, 46]. However, the accuracy of these derived metrics depends heavily on the initial accuracy of the HPE. Furthermore, to enhance postural analysis and ensure clinical relevance, these systems require a baseline for comparison [32, 33, 35]. This highlights a significant challenge in the field: the need for established normative posture data to differentiate between healthy variation and pathological deviation [8, 18, 32, 47]. The development of large, diverse, and accurately labelled datasets is therefore as important as the development of the AI models themselves, as they provide the necessary foundation for training robust models and establishing a valid reference for assessment [18, 35, 36]. A comparative overview of the primary data acquisition modalities, their associated AI model types, and key characteristics is provided in Table 2.

**Table 2:** Detection technologies at a glance

Sensor/modality	AI/model type	Strengths	Limitations
2D RGB cameras	2D human pose estimation (CNN-based)	Low-cost, accessible, markerless; suitable for tele-rehab	Prone to perspective distortion; limited biomechanical precision
Depth-sensing cameras (Kinect, RealSense)	3D human pose estimation (multi-view/depth-based DL)	Provides true 3D data; higher biomechanical accuracy	Requires specific hardware; environment constraints
IMUs	ML/DL models for orientation and joint angle estimation	Robust to lighting/occlusion; captures dynamic movements	Requires user compliance with wearing sensors
Lighthouse-based systems	ML/DL models for 3D skeletal reconstruction	Sub-centimeter precision; full-body tracking; relatively low cost	Requires calibrated space; continuous line-of-sight; limited portability
Wearable smart textiles	ML algorithms for strain/pressure data interpretation	Comfortable, continuous monitoring; unobtrusive	Still experimental; limited large-scale validation
Radar/pressure sensors	Pattern recognition, anomaly detection ML models	Non-visual, privacy-preserving; useful in cluttered environments	Limited resolution; fewer studies in clinical contexts

2D, Two-dimensional; 3D, Three-dimensional; CNN, Convolutional neural network; DL, Deep learning; IMU, Inertial measurement unit; ML, Machine learning; RGB, Red, green and blue

## Benchmarking detection accuracy and system performance

The proliferation of AI-based technologies for postural detection necessitates a rigorous and critical evaluation of their accuracy, reliability, and clinical validity [27]. While AI models can achieve technically impressive results in controlled environments, their true value is determined by their performance in real-world scenarios and their ability to provide information that is both accurate and clinically meaningful [27, 48]. Benchmarking these systems involves comparing their outputs against established “gold standard” measurements and assessing their performance across diverse populations and conditions [27]. A fundamental aspect of benchmarking is validation against a trusted ground truth. In clinical research, this often involves comparing the AI-derived postural measurements against those obtained from radiographic imaging, which has long been a standard for assessing skeletal alignment [21, 27]. For dynamic movements, the gold standard is typically a laboratory-grade, marker-based 3D motion capture system [49]. These systems provide highly precise sub-millimeter tracking of body kinematics and serve as an ideal reference for quantifying the error of markerless, vision-based AI systems [35, 36, 50]. Studies that undertake such direct comparisons are essential for establishing the concurrent validity of new technologies. For example, research assessing AI-based software for measuring cervical and lower-limb alignment against radiographic data provides crucial evidence regarding the tool’s reliability and interchangeability with traditional methods [27, 51]. However, the literature indicates that a straightforward correlation is not always sufficient [52]. CV applications in sports biomechanics, for instance, have demonstrated widely varying levels of accuracy depending on the specific task, environment, and model used [17, 53, 54]. Accuracy can be influenced by a multitude of factors, including:

- *Model and modality* The choice between a 2D or 3D HPE model fundamentally impacts the potential accuracy of biomechanical calculations. 3D models are inherently more capable of capturing complex spatial relationships, but their performance can be dependent on specialized hardware [28, 55].
- *Environmental conditions* Factors such as lighting, background clutter, and camera angle can significantly degrade the performance of vision-based systems [52, 56].
- *Subject-specific factors* The subject’s clothing (e.g., loose-fitting garments that obscure body contours), body mass index, and skin tone can affect the key point detection accuracy of models, particularly if the training data was not sufficiently diverse [56].
- *Occlusion* When parts of the body are hidden from the camera's view (e.g., one arm blocking the torso), the AI model must infer the occluded key points' locations, which can introduce significant error [32, 52].

These factors contribute to a critical gap between performance in a controlled laboratory setting and performance “in the wild” [33]. An AI model trained on a curated dataset of athletes performing specific movements might fail to generalize to an elderly individual with atypical movement patterns or a factory worker in a visually complex industrial environment [57]. This is why developing robust AI techniques for full-body pose estimation is an area of active research, aiming to create models that are resilient to these real-world challenges [28].

Ultimately, technical accuracy metrics, such as the mean per joint position error used in computer science research, are not the sole arbiters of a system's utility [21, 58]. A system can be technically precise but clinically irrelevant. A crucial, and often overlooked, aspect of performance evaluation is the assessment of whether AI-derived postural measurements agree with broader biomechanical and symptomatic contexts [27, 59]. For a clinician, a tool is only useful if its outputs correlate with patient-reported outcomes, functional limitations, or risk of injury [19, 60]. A system that detects a 2-degree spinal deviation is of little value if that deviation is not associated with pain or functional impairment [61]. Therefore, the future of benchmarking must move beyond simple error metrics and focus on establishing clinical validity [27]. This requires studies that not only measure the agreement between AI and gold standards but also investigate how AI-driven insights can be used to predict clinical outcomes, guide therapeutic decisions, and improve patient health. Establishing this connection is paramount for the responsible and effective integration of AI into postural management.

## Evaluating the efficacy of AI-based postural correction interventions

Following the critical analysis of AI for postural detection, this chapter transitions from assessment to intervention [62]. The true clinical value of identifying postural deviations lies in the ability to effectively correct them, improve function, and mitigate associated health risks [5]. The integration of AI into therapeutic strategies represents a paradigm shift from conventional, often intermittent and supervised, postural correction methods to dynamic, personalized, and continuous interventions [25, 63, 64]. This chapter evaluates the efficacy of these emerging AI-based postural correction systems. It critically examines three core domains: the application of real-time biofeedback for active correction, the development of AI-guided therapeutic programs within TR frameworks, and the current landscape of clinical evidence supporting their effectiveness [65, 66]. By dissecting these areas, the chapter aims to provide a comprehensive understanding of the current capabilities, therapeutic potential, and empirical validation of AI in actively managing and improving human posture [64, 67].

## Real-time biofeedback and correction systems

Real-time biofeedback is a cornerstone of modern motor learning and rehabilitation, providing individuals with immediate information about physiological processes that are typically unconscious [65]. In the context of postural management, this involves making the user aware of their body alignment and movement patterns as they occur, facilitating self-correction [27]. The advent of AI, coupled with ubiquitous sensing technologies, has profoundly enhanced the sophistication and accessibility of biofeedback systems [62]. These systems create a closed-loop interactive environment where the AI acts as a perpetual, vigilant coach, transforming postural correction from a passive exercise into an active, engaged process [64, 68]. AI-driven biofeedback systems operate on a detect–inform–correct cycle. Leveraging the HPE models discussed previously, these systems first analyze data streams from sources like cameras or wearable sensors to identify deviations from an ideal or personalized postural baseline [28, 33]. Once a postural error is detected, such as slouching at a table, an asymmetrical posture, or incorrect form during exercise, the system provides immediate feedback [22, 23, 62]. This feedback can be rendered through various sensory channels:

### Visual feedback

This is the most common modality, where an on-screen avatar mirrors the user’s movements, with incorrect joint angles or body segments highlighted in red. The interface may display corrective cues, such as arrows indicating the required direction of movement or a “posture score” that fluctuates in real time. This visual representation allows users to directly see the discrepancy between their perceived and actual posture [22, 69].

### Auditory feedback

Systems can use simple tones, chimes, or spoken commands (e.g., “Sit up straight”, “Shoulders back”) to alert the user to a postural fault. This modality is particularly useful for applications where the user’s visual attention is focused elsewhere, such as during office work or driving [70].

### Haptic feedback

This involves physical sensations, typically vibrations delivered by wearable devices placed on the torso or specific body segments. A gentle buzz can serve as a discreet, private reminder to correct posture without requiring visual or auditory engagement, making it ideal for social or professional settings [71].

The main aim of this immediate feedback is to improve proprioception—the body’s inherent sense of its position in space. By consistently identifying postural errors, AI systems train the user to become more aware of their own body alignment, gradually recalibrating their natural “default” posture [71, 72]. This process of active correction empowers patients, allowing them to take direct control over their posture and muscle activity [12, 23]. Modern systems often integrate these feedback mechanisms into tools that assist both the patient and the clinician, providing a continuous stream of data for monitoring progress and adjusting therapeutic strategies [73].

Furthermore, the application of AI-based biofeedback extends beyond static postures to dynamic movements and therapeutic exercises [26, 65]. Systematic reviews of ML applications have shown that AI can effectively detect key postural errors during exercises, serving a critical role in injury prevention [68]. For instance, a system can monitor a user performing a squat and provide real-time cues if their knees drift inward (knee valgus) or their back rounds excessively [51, 60]. This guidance ensures that exercises are performed safely and effectively, maximizing therapeutic benefit while minimizing the risk of secondary injury [19, 22].

The integration of structured biofeedback is particularly crucial in managing chronic conditions like low back pain, where it empowers users to consciously modify their posture and movement habits throughout the day [74]. These systems actively engage patients in their own recovery, representing a significant evolution from traditional rehabilitation paradigms that rely heavily on periodic clinical supervision [66, 74]. The capacity for AI to provide this level of detailed, real-time guidance is a fundamental component of its therapeutic promise in postural management [64, 65].

## AI-guided therapeutic exercise and TR

While real-time biofeedback focuses on immediate correction, AI-guided therapeutic programs leverage this technology within a broader, long-term rehabilitation framework, often delivered through TR platforms [65, 66]. Conventional TR often relies on simple video conferencing and patient self-reporting, lacking objective, real-time analysis of exercise execution [75]. The integration of AI fundamentally elevates these platforms from passive communication tools into sophisticated, interactive, and personalized therapeutic systems [24, 65, 66] by addressing key limitations of conventional methods. An AI-guided TR program for postural correction typically encompasses a comprehensive, multi-stage process. It begins with a remote assessment, where the AI system uses a device’s camera for markerless motion analysis, a significant advantage over systems requiring costly or cumbersome wearable sensors [32, 35, 66]. Based on this initial evaluation, the AI can help generate a personalized therapeutic program. This moves beyond the one-size-fits-all exercise sheets common in conventional remote care, by tailoring routines to address the individual’s specific deficits identified through the AI's objective analysis [12, 63, 64, 66]. The core of the intervention lies in the guided execution of these exercises. The system uses CV to monitor the patient’s performance, providing real-time feedback on form. This is a critical advancement over conventional TR, where form correction is limited to the therapist's view during a scheduled call and is absent during independent practice [65, 66]. Furthermore, the AI’s role extends beyond what is possible with simple sensors: while an IMU might count repetitions, AI can qualitatively assess movement, detecting subtle errors like asymmetrical weight shift or improper spinal alignment that are invisible to repetition-counting algorithms [51, 60, 68, 76]. This data is logged automatically, creating a detailed record of adherence and quality of performance [66, 77, 78]. One of the most significant advantages of this approach is its capacity for dynamic personalization and adaptation. Clinicians can leverage the system to remotely monitor progress and adjust plans [24, 77–80]. Critically, the AI itself can be designed to automatically progress exercise difficulty based on performance, creating an adaptive loop that is impossible in static, conventional home exercise programs. This creates a responsive rehabilitation experience that more closely mirrors the personalization of in-person, one-on-one therapy [24, 80, 81]. A direct comparison of the core capabilities and limitations of conventional versus AI-based systems for postural management is provided in Table 3.

**Table 3:** A comparison of conventional and AI-based approaches in postural biofeedback and tele-rehabilitation

Feature/capability	Conventional (non-AI) systems	AI-based systems	AI’s added value/mechanism
Posture detection and assessment	Relies on fixed, rule-based thresholds from single sensors (e.g., IMU tilt angle); visual inspection via video call	Uses DL-based HPE for markerless, multi-joint kinematic analysis from standard cameras	Holistic and granular analysis: Moves beyond single-angle measurement to a comprehensive skeletal model, enabling detection of complex, compound postures and subtle asymmetries that are difficult to perceive visually
Feedback specificity	Generic, threshold-triggered alerts (e.g., a vibration when slouching); qualitative verbal feedback from a therapist during sessions	Specific, actionable, real-time instructions (e.g., “rotate right shoulder back”, “reduce knee valgus”) via visual, auditory, or haptic cues	Precision guidance: Provides context-aware feedback on the specific nature and direction of the error, facilitating more effective motor learning and self-correction
Exercise quality monitoring	Limited to simple metrics like repetition count (from accelerometers); qualitative form assessment is limited to scheduled therapy sessions	Automated, real-time qualitative assessment of movement form, technique, and compensation patterns during every exercise session	Objective quality control: Provides therapist-level oversight of exercise fidelity remotely, ensuring exercises are performed safely and effectively, which is a major limitation of conventional independent home exercise
Personalization and adaptation	Static exercise prescriptions; progression requires manual re-assessment and adjustment by a clinician	Dynamic personalization; can automatically adapt exercise difficulty and type based on continuous performance data	Adaptive intervention: Creates a closed-loop system that maintains the optimal challenge for each patient, improving engagement and outcomes, a feature impossible with static paper-based or generic digital programs
Data modality and accessibility	Primarily reliant on wearable sensors (IMUs), which require user compliance and correct placement	Primarily markerless, using ubiquitous hardware (RGB cameras); can optionally fuse with wearable data	Increased accessibility and usability: Lowers the barrier to entry by eliminating the need for specialized wearable sensors, facilitating deployment in home environments
Primary limitation	Inflexible, can provide context-inappropriate alerts; limited in the complexity of movements it can analyze; requires clinician time for progression	Requires large, diverse datasets for training; potential performance issues in suboptimal environments (e.g., poor lighting); “black box” nature can reduce interpretability	

AI, Artificial intelligence; DL, Deep learning; HPE, Human pose estimation; IMU, Inertial measurement unit; RGB, Red, green and blue

## Evidence from clinical trials and comparative studies

The technological sophistication and theoretical promise of AI-based postural correction interventions are compelling; however, their clinical value must ultimately be substantiated by rigorous empirical evidence [27]. The evaluation of efficacy requires moving beyond technical performance metrics to demonstrate tangible improvements in patient outcomes, such as reduced pain, enhanced function, and long-term postural improvement [25, 73]. The body of evidence in this domain is growing, characterized by an increasing number of systematic reviews and a gradual shift toward comparative clinical trials, yet it remains a field in development [65, 68, 82, 83]. The current evidence base is largely composed of feasibility studies, pilot trials, and systematic reviews that collate findings from smaller-scale research. These reviews confirm the technical viability and high user acceptance of AI-driven systems. For example, a review of remote sensor-based monitoring for low back pain highlights the effectiveness of using structured biofeedback and wearable sensor technologies to help users modify their posture, with positive implications for rehabilitation care [84]. Similarly, the use of TR platforms equipped with biofeedback is frequently cited as a promising approach for actively engaging patients and personalizing care, supported by a growing number of studies across various musculoskeletal and neurological conditions [65]. These reviews lay a crucial foundation by establishing that the technology works as intended and is generally well received by patients and clinicians. However, the more critical question is whether these novel interventions are superior to, or at least as effective as, conventional therapy [80, 83]. Answering this requires comparative studies, particularly randomized controlled trials (RCTs), which are considered the gold standard for clinical evidence. Research in this area is beginning to emerge. For instance, some investigations include clinical trials that directly compare TR programs with conventional, in-person therapy [85]. These studies are vital for establishing clinical non-inferiority or superiority. While many are still in their early stages, they represent a critical step toward validating these technologies for widespread clinical adoption. The field is increasingly emphasizing the need for high-quality systematic reviews and meta-analyses to synthesize the results of these trials and provide a clearer picture of overall efficacy. A significant challenge in this area is the heterogeneity of the interventions and outcome measures. AI-based systems vary widely in their hardware (e.g., webcam, depth sensor, IMUs), software algorithms, feedback modalities, and the structure of their therapeutic programs. Likewise, studies may measure different outcomes, ranging from objective biomechanical parameters (e.g., spinal curvature angles) to patient-reported outcomes like pain, disability scores, and quality of life. This variability makes it difficult to compare results across studies and draw definitive conclusions. For example, literature exploring innovations in telemedicine and remote monitoring often focuses on outcomes related to quality of life as a key indicator of an intervention’s success, acknowledging that clinical efficacy must translate to meaningful benefits for the patient [24, 65, 66].

In summary, the evidence for the efficacy of AI-based postural correction interventions is promising but not yet conclusive. There is strong foundational evidence supporting their feasibility and capacity to deliver personalized biofeedback and remote therapy [64, 79]. The field is actively engaged in building a more robust evidence base through systematic reviews and the execution of comparative clinical trials. However, a significant gap remains in the form of large-scale, long-term RCTs that can definitively establish the clinical and cost-effectiveness of these AI systems compared to standard care across diverse patient populations. Future research must prioritize standardized protocols and clinically meaningful outcomes to bridge the gap between technological innovation and evidence-based clinical practice. The diverse landscape of AI-based postural correction interventions, along with a summary of their associated clinical evidence, is presented in Table 4.

**Table 4:** Correction interventions in clinical studies

Intervention type	Delivery modality	Target population	evidence/clinical findings
Visual biofeedback	On-screen avatar, posture score, corrective cues	Office workers, rehab patients, athletes	Improves awareness, helps retraining, but adherence varies
Auditory biofeedback	Chimes, tones, voice prompts	Drivers, office workers	Effective for attention-demanding tasks; limited trials
Haptic feedback (wearables)	Vibration motors embedded in wearable devices	Daily use in professional or social settings	Discrete reminders improve compliance; promising pilot trials
AI-guided exercise (tele-rehabilitation)	Remote platforms with AI-monitored exercises	MSK disorders, neurological rehab, older adults	RCTs show comparable or improved outcomes vs conventional rehab
Exoskeleton-assisted correction	Robotic assistive devices with adaptive AI control	Severe spinal deformities, stroke rehab, mobility-impaired patients	Strong lab-based evidence; limited large-scale clinical trials

AI, Artificial intelligence; MSK, Musculoskeletal; RCT, Randomized controlled trial

## Clinical applicability, integration, and future challenges

**Fig. 2:**
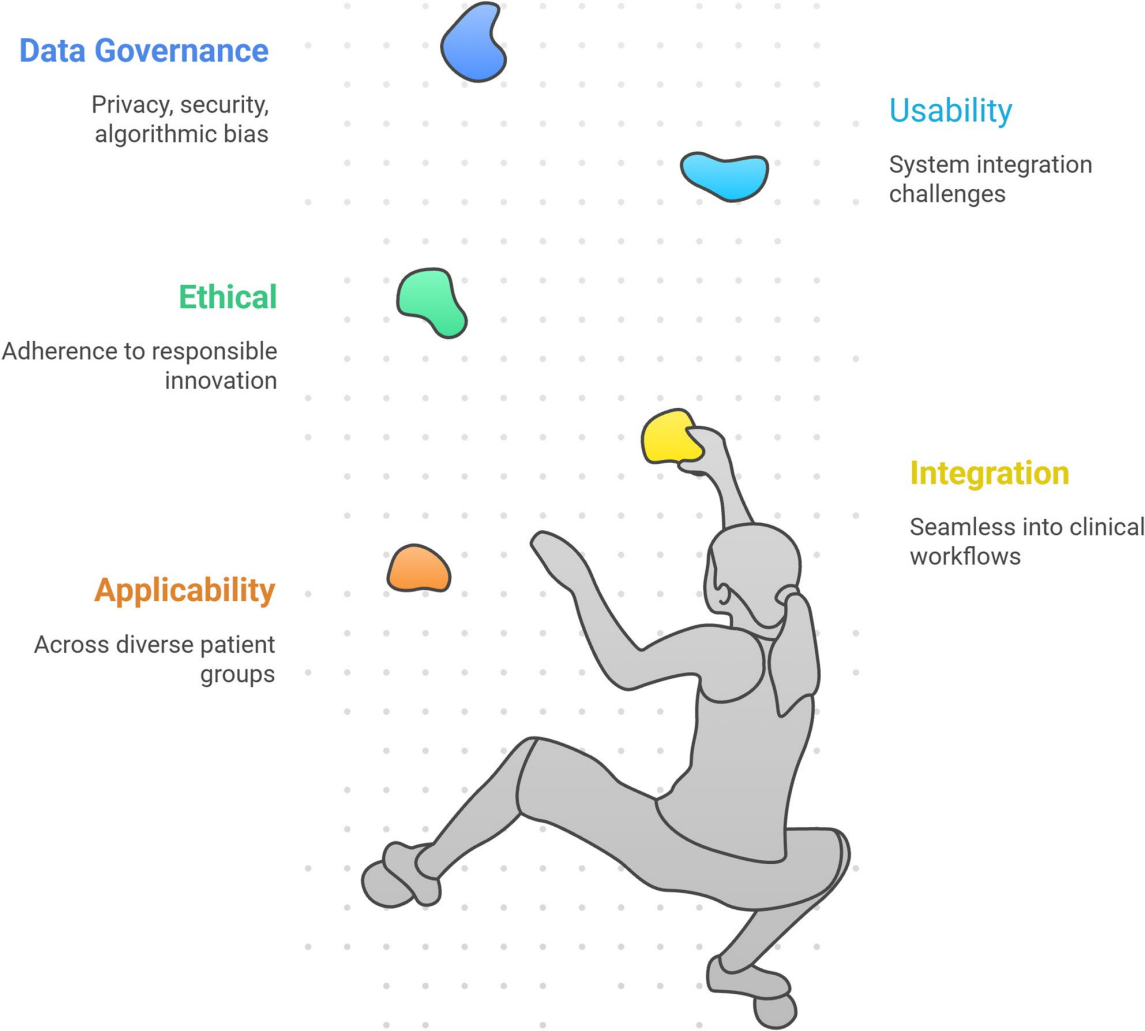
Clinical applicability, integration, and future challenges

The translation of AI from a research concept into a clinically viable tool for postural management is contingent on overcoming significant practical, usability, and ethical hurdles (Fig. 2). While the technical capabilities for AI-driven postural detection and correction are advancing rapidly, their real-world impact is ultimately determined by their applicability across diverse patient groups, seamless integration into clinical workflows, and adherence to principles of responsible innovation [86, 87]. This chapter critically examines these dimensions, exploring the current landscape of clinical applications, the challenges related to usability and system integration, and the pressing ethical considerations that must be addressed to ensure safe, equitable, and effective deployment of these technologies.

## Application across diverse clinical populations

The potential of AI in postural management extends across a wide spectrum of clinical populations, each with unique needs and challenges. The adaptability of AI algorithms allows for the development of tailored interventions that can address specific postural deficits, moving beyond a one-size-fits-all approach. For instance, intelligent systems are being designed to address the complex needs of non-ambulatory individuals, such as monitoring the sitting posture of wheelchair users to detect and prevent anomalies that can lead to pressure sores and musculoskeletal deformities [64, 73, 88]. By providing continuous, non-invasive monitoring, these systems offer a proactive solution for a population at high risk for secondary complications arising from prolonged static postures. In the realm of rehabilitation for common MSDs, AI is demonstrating significant promise [88]. For conditions like low back pain, ML tools have been integrated into assessment protocols to analyze movement patterns, predict rehabilitation outcomes, and guide clinical decision-making [23, 74]. This data-driven approach allows therapists to personalize treatment plans based on objective metrics of a patient’s functional status and progress. Similarly, in spine care, AI is being leveraged to identify early signs of postural and gait abnormalities that may predispose individuals to injury or chronic conditions [64]. By flagging subtle deviations that might be missed during routine clinical observation, such systems can facilitate early intervention and improve treatment adherence and recovery outcomes [64, 79]. For specific spinal deformities, such as scoliosis, AI-enhanced wearable devices are emerging as tools to promote better posture awareness and supplement traditional bracing and exercise regimens, with some innovations being validated through clinical trials [89]. One of the most impactful areas of application is in geriatric care, where maintaining postural control is critical for preserving independence and preventing falls [90]. A randomized controlled trial involving older adults with sarcopenia successfully validated an AI-based remote rehabilitation program using 3D human posture estimation. The study found that this TR approach led to significant improvements in dynamic postural control, demonstrating its effectiveness and practicality for delivering care outside of traditional clinical settings (despite usability challenges like tech familiarity, mitigated by user-friendly designs and brief onboarding (85% adherence rate)). Such systems empower older adults to engage in therapy from the comfort of their homes, overcoming barriers related to mobility and transportation [91, 92]. As the technology matures, its application is expected to expand to other populations, including pediatric patients with developmental disorders, athletes seeking to optimize performance and prevent injury, and individuals with neurological conditions such as Parkinson’s disease or stroke, where postural instability is a primary symptom.

## Usability, user adherence, and system integration

For any AI-driven postural management system to achieve clinical success, it must be both usable for the end-user and seamlessly integrable into existing healthcare infrastructures. High detection accuracy and sophisticated algorithms are rendered ineffective if the technology is cumbersome, unintuitive, or disruptive to patient and clinician workflows [23, 28]. Usability is a cornerstone of effective implementation, as a user-friendly application is more likely to facilitate active patient participation in postural correction exercises and provide clinicians with accessible data analysis capabilities [78]. Systems should require minimal setup, provide clear and actionable feedback, and be robust enough to function reliably in real-world environments, which are often less controlled than a research laboratory [91]. User adherence is intrinsically linked to usability and is a critical determinant of long-term therapeutic outcomes. Traditional postural correction programs often suffer from low adherence due to the repetitive nature of exercises and the lack of immediate, tangible feedback [78]. AI-based systems can address this challenge by incorporating elements of gamification, personalized goal setting, and progress visualization. This can transform monotonous rehabilitation routines into engaging experiences, thereby improving motivation and treatment adherence [17, 66]. The ability of AI to provide continuous monitoring and real-time feedback creates a dynamic interactive loop that reinforces correct movement patterns and empowers patients to take an active role in their own care, a factor that is particularly important for enhancing rehabilitation outcomes [24, 86]. Beyond patient-facing usability, the successful integration of these systems into clinical practice presents a formidable challenge [93]. AI tools must not function as isolated data silos; instead, they must interoperate with established health information systems, such as electronic health records. This integration is crucial for enabling clinicians to gain a comprehensive understanding of the patient's progress and for incorporating AI-generated insights into the overall treatment plan. AI should serve as a decision-support tool, augmenting the clinician’s expertise by providing objective, quantitative data that can help predict rehabilitation outcomes or guide clinical decision-making [25, 81, 93]. The goal is to streamline workflows, reduce administrative burden, and equip clinicians with deeper insights, rather than adding another layer of complexity to their practice. The transition from standalone research prototypes to fully integrated, clinical-grade systems will require significant collaboration between technology developers, healthcare providers, and IT professionals to ensure that these powerful tools can be deployed effectively and at scale.

## Ethical considerations and responsible innovation

The integration of AI into postural management, like any other health-related technology, carries significant ethical responsibilities that must be proactively addressed. Fostering responsible innovation requires a commitment to ethical best practices throughout the technology's lifecycle, from data collection and algorithm development to clinical deployment and long-term monitoring [87]. A primary concern is the privacy and security of the highly sensitive personal health data collected by these systems. Visual data from cameras, kinematic data from sensors, and diagnostic information must be protected through robust encryption, secure storage, and stringent access controls to prevent unauthorized access and misuse. Patients must provide genuine informed consent, which entails a clear understanding of what data is being collected, how it will be used, who will have access to it, and the inherent limitations of the AI system. Another critical ethical challenge is the potential for algorithmic bias. AI models learn from the data they are trained on, and if this data is not representative of the broader population, the resulting algorithm may perform less accurately for underrepresented groups based on factors such as age, gender, ethnicity, body type, or disability. For example, a posture detection model trained primarily on one demographic may fail to accurately assess posture in others, leading to misdiagnosis and perpetuating health inequities. To mitigate this risk, developers must prioritize the curation of large, diverse, and well-annotated datasets and conduct rigorous validation to ensure the system's fairness and equity across different populations. Furthermore, the question of accountability in the event of an AI-driven error is complex. If a system fails to detect a critical postural issue or provides a harmful corrective recommendation, determining liability, whether it lies with the software developer, the healthcare institution, or the supervising clinician, is a legal and ethical minefield. This ambiguity underscores the importance of maintaining human oversight in the clinical loop. AI should be positioned as a tool that augments, rather than replaces, the professional judgment of a trained clinician. The “black box” nature of some complex AI models also poses a challenge; a lack of transparency into how a model arrives at a conclusion can erode trust and hinder a clinician's ability to critically evaluate its output. Therefore, a push towards explainable AI is vital in the medical domain. Responsible innovation in this field demands a multi-stakeholder approach, involving ethicists, clinicians, patients, and regulators in the design and governance of these technologies to ensure they are developed and deployed in a manner that is safe, effective, equitable, and worthy of public trust. The success of AI-based TR and home monitoring programs, for example, is predicated on a foundation of trust that can only be built through such a conscientious approach [86, 87].

## Conclusions

This review confirms the significant potential of AI to revolutionize postural management by enabling objective, continuous assessment and personalized, real-time correction. AI-driven systems have demonstrated high accuracy in detecting postural deviations and show promise in improving patient engagement and rehabilitation outcomes through biofeedback and TR. However, a substantial gap remains between technical potential and real-world clinical utility. Widespread adoption is hindered by challenges including limited generalizability of AI models beyond controlled lab settings, a lack of large-scale clinical trials proving long-term efficacy, and practical barriers related to usability, data privacy, and integration into healthcare workflows. To realize AI's transformative potential, future efforts must prioritize robust clinical validation in diverse populations, user-centered design to ensure practicality and adherence, and a steadfast commitment to ethical, responsible innovation. By addressing these challenges, AI can evolve from a promising tool into an indispensable asset for proactive, equitable, and effective postural care.

## Data Availability

Data sharing is not applicable to this article as no datasets were generated during the current study.

## Abbreviations

2D: Two-dimensional
3D: Three-dimensional
AI: Artificial intelligence
CNN: Convolutional neural network(s)
CV: Computer vision
DL: Deep learning
HPE: Human pose estimation
IMU: Inertial measurement unit(s)
ML: Machine learning
MSDs: Musculoskeletal disorders
RCT: Randomized controlled trial
RGB: Red, green, and blue
TR: Tele-rehabilitation
